# Impact of family networks on uptake of health interventions: evidence from a community‐randomized control trial aimed at increasing HIV testing in South Africa

**DOI:** 10.1002/jia2.26142

**Published:** 2023-08-20

**Authors:** Keletso Makofane, Hae‐Young Kim, Eric Tchetgen Tchetgen, Mary T. Bassett, Lisa Berkman, Oluwafemi Adeagbo, Nuala McGrath, Janet Seeley, Maryam Shahmanesh, H. Manisha Yapa, Kobus Herbst, Frank Tanser, Till Bärnighausen

**Affiliations:** ^1^ Department of Biostatistics, Epidemiology and Informatics University of Pennsylvania Philadelphia United States; ^2^ Department of Population Health New York University Grossman School of Medicine New York New York USA; ^3^ Africa Health Research Institute Kwa‐Zulu Natal South Africa; ^4^ Department of Statistics and Data Science, The Wharton School University of Pennsylvania Philadelphia Pennsylvania USA; ^5^ FXB Center for Health and Human Rights Harvard University Boston Massachusetts USA; ^6^ Harvard Center for Population and Development Studies Harvard University Cambridge United States; ^7^ Department of Social Statistics and Demography University of Southampton Southampton UK; ^8^ Department of Global Health and Development London School of Hygiene & Tropical Medicine London UK; ^9^ Institute for Global Health University College London London UK; ^10^ Kirby Institute for Infection and Immunity University of New South Wales Sydney New South Wales Australia; ^11^ Centre for Epidemic Response and Innovation, School for Data Science and Computational Thinking Stellenbosch University Stellenbosch South Africa; ^12^ School of Nursing and Public Health University of Kwa‐Zulu Natal Durban South Africa; ^13^ Centre for the AIDS Programme of Research in South Africa (CAPRISA) University of Kwa‐Zulu Natal Durban South Africa; ^14^ Heidelberg Institute of Global Health, Faculty of Medicine and University Hospital University of Heidelberg Heidelberg Germany

**Keywords:** HIV epidemiology, testing, social networks, randomized controlled trial (RCT), AHRI, social epidemiology

## Abstract

**Introduction:**

While it is widely acknowledged that family relationships can influence health outcomes, their impact on the uptake of individual health interventions is unclear. In this study, we quantified how the efficacy of a randomized health intervention is shaped by its pattern of distribution in the family network.

**Methods:**

The “Home‐Based Intervention to Test and Start” (HITS) was a 2×2 factorial community‐randomized controlled trial in Umkhanyakude, KwaZulu‐Natal, South Africa, embedded in the Africa Health Research Institute's population‐based demographic and HIV surveillance platform (ClinicalTrials.gov # NCT03757104).

The study investigated the impact of two interventions: a financial micro‐incentive and a male‐targeted HIV‐specific decision support programme. The surveillance area was divided into 45 community clusters. Individuals aged ≥15 years in 16 randomly selected communities were offered a micro‐incentive (R50 [$3] food voucher) for rapid HIV testing (intervention arm). Those living in the remaining 29 communities were offered testing only (control arm). Study data were collected between February and November 2018.

Using routinely collected data on parents, conjugal partners, and co‐residents, a socio‐centric family network was constructed among HITS‐eligible individuals. Nodes in this network represent individuals and ties represent family relationships. We estimated the effect of offering the incentive to people with and without family members who also received the offer on the uptake of HIV testing. We fitted a linear probability model with robust standard errors, accounting for clustering at the community level.

**Results:**

Overall, 15,675 people participated in the HITS trial. Among those with no family members who received the offer, the incentive's efficacy was a 6.5 percentage point increase (95% CI: 5.3−7.7). The efficacy was higher among those with at least one family member who received the offer (21.1 percentage point increase (95% CI: 19.9−22.3). The difference in efficacy was statistically significant (21.1–6.5 = 14.6%; 95% CI: 9.3−19.9).

**Conclusions:**

Micro‐incentives appear to have synergistic effects when distributed within family networks. These effects support family network‐based approaches for the design of health interventions.

## 1 INTRODUCTION

Though family relationships crucially determine health and wellbeing, their role in shaping the uptake of individual health interventions is not well‐understood. Using family network data from a large population‐based cohort, we quantified the degree to which the efficacy of a randomized individual‐level health intervention—a financial incentive for HIV testing—is shaped by its pattern of distribution among family members.

Improving testing programmes can increase access to anti‐retroviral therapy (ART) which effectively eliminates HIV transmission at the individual level [1] and has substantially reduced population incidence [2, 3, 4]. Recognizing the uneven distribution of risk and access to services in so‐called “generalized epidemics” [5], recent global public health guidance advocates multiple strategies for testing in these settings [6, 7]. Interventions that leverage personal networks are among the most effective [6, 7]. For instance, distributing HIV self‐test kits to men through their sexual, romantic and other social relationships has been shown to improve the acceptability and uptake of testing [8, 9, 10, 11]. Unrelated to networks but also effective are testing interventions that utilize financial incentives [12, 13, 14].

Through a post‐hoc analysis of Home‐Based Intervention to Test and Start (HITS) study data, we quantified how the effectiveness of a financial incentive for HIV testing changes depending on whether it is offered to an individual or offered to an individual along with family members. HITS, which was conducted in South Africa, investigated the effects on HIV testing and linkage to care of a ZAR 50 (USD 3) incentive and a male‐targeted HIV‐specific decision support programme. We previously reported that among men, the uptake of HIV testing increased from 17.1% in the standard of care to 27.5% in the financial incentives arm (risk ratio = 1.55, 95% CI: 1.31−1.82) [12].

We build on this finding by testing the hypothesis that, for a given individual, the effectiveness of the financial incentive is augmented by offering incentives to family members prior to, or at the same time as, the individual. For many South Africans, resources are shared among extended family across different households [15]. It is possible, therefore, that over the course of the HITS trial, family members influenced each other's HIV testing behaviour in order to maximize receipt of incentives.

## 2 METHODS

### 2.1 Setting and participants

HITS is a community‐randomized controlled trial in the Hlabisa sub‐district of the uMkhanyakude district—a rural region of northern KwaZulu‐Natal with a high HIV burden and unemployment [16, 17, 18]. It is nested in the Africa Health Research Institute's population‐based demographic and HIV surveillance platform which follows 140,000 residents living in an area of 845 km^2^ [16]. As part of annual routine HIV surveillance, trained field workers visit all households and record demographic information, including parents, co‐residents and conjugal partners of each household member. During visits, all residents aged 15 years or older are offered home‐based rapid HIV testing.

Individuals were eligible for HITS if they were 15 years or older at the time of the surveillance visit, resided within the surveillance area, agreed to participate in annual HIV surveillance and provided written informed consent for trial participation. Individuals were not eligible to participate in the trial if they refused to participate in HIV surveillance, reported being already on ART or were mentally or physically unable to provide consent. The study is registered at the U.S. National Institute for Health's ClinicalTrials.gov (# NCT03757104). Further details are available in earlier publications [19].

### 2.2 Randomization and masking

**Figure 1:**
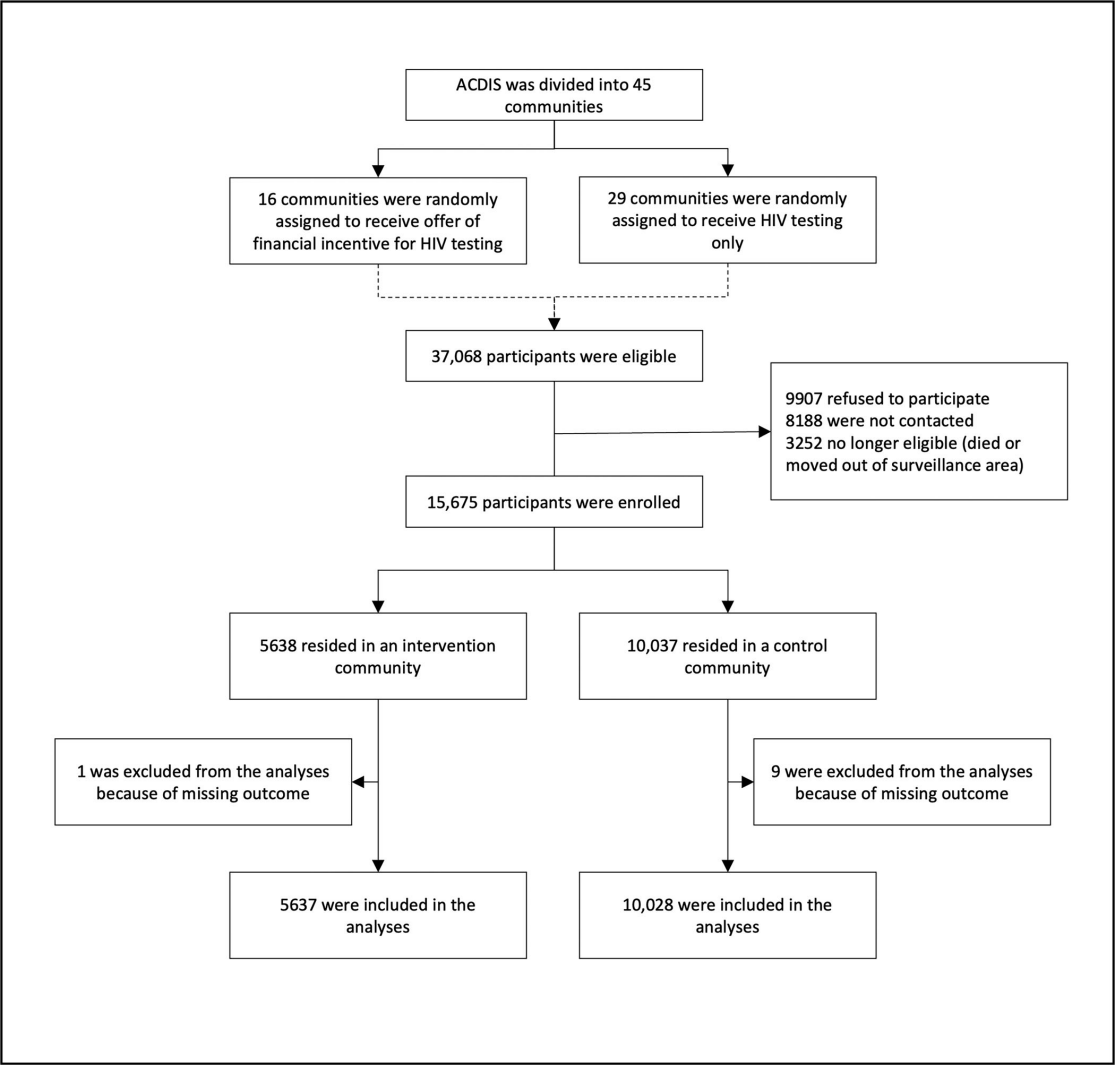
Flow diagram for HITS trial.

The HITS study investigated two interventions: a financial micro‐incentive for HIV testing and a male‐targeted HIV‐specific decision support programme [12, 19]. The surveillance area was divided into 45 community clusters which were randomized to study interventions using a 2 × 2 factorial design, permitting each intervention to be assessed separately. Interventions were delivered between February and November 2018. We consider the effect of the micro‐incentive alone since the other study intervention was restricted to men, whereas our analysis includes all HITS participants (see Figure 1).

The 45 communities were grouped into four strata based on baseline HIV incidence rates among women aged 15–30 years. The intervention arm consisted of four randomly selected communities from each of the four strata (16 communities total). The control arm consisted of the remainder of the communities in each stratum (29 communities total). The study was an open‐label trial.

### 2.3 Consent and intervention

Only residents who agreed to participate in annual AHRI HIV surveillance were eligible to participate in HITS. Residents were asked for their consent at the study visit. Those who consented to AHRI HIV surveillance were then asked for their consent to participate in the HITS study.

Those who were eligible for and consented to participate in HITS were enrolled. Those who resided in control communities were offered rapid HIV testing per the HIV surveillance protocol. Those in intervention communities were offered a micro‐incentive conditional on undergoing home‐based rapid HIV testing during the study visit. The micro‐incentive was a food voucher valued at ZAR 50 (∼USD 3), which was redeemable at a local supermarket [19].

### 2.4 Social network

A socio‐centric family network was constructed among HITS‐eligible individuals using routinely collected surveillance data. Nodes in this network represent individuals. Three kinds of ties were added between the nodes: first‐degree relatives (parents, children and all conjugal partners of each participant), second‐degree relatives (the first‐degree relatives of first‐degree relatives) and co‐resident relatives (individuals who ever resided in the same household as the participant and who were not tenants or domestic workers in that household). Below, we refer to members of each person's personal family network (i.e. the egocentric network) simply as “family members.”

Family members of residents are only recorded if they ever resided in the surveillance area. For most individuals, it was possible to identify at least one family member—only 2.7% (424/15,675) of HITS participants were not linked with any other residents. Because surveillance began in 2000, older residents were less likely to be observed at the same time as their parents. Among the records of individuals aged 15–25, 15.0% (2321/15,458) were not linked to their mother's record and 48.3% (7471/15,458) were not linked to their father's. Among those over 55 years of age, these proportions were 87.9% (4795/5458) and 97.3% (5310/5458), respectively. Missing linkages between participants and their parents indicate that their parents were not eligible for the study.

### 2.5 Measures

The outcome of interest was individual uptake of rapid HIV testing at the study visit. Exposures of interest were individual offer of financial incentive (“individual offer”) and family offer of incentive (“family offer”). For each participant, family offer was defined as the count of family members who were offered the financial incentive prior to or on the same day as the participant's own study visit. Network size was defined as the count of family members.

### 2.6 Analysis

We calculated sample characteristics, examined patterns of network connections between communities and described the composition of network connections.

For the primary analysis, we examined heterogeneity in the effect of the individual offer on HIV testing uptake across strata defined by the dichotomized family offer (≥1 vs. 0). We fitted a linear probability regression model with a two‐way interaction encoding the extent to which the causal effect of individual offer is modified by dichotomized family offer. (See Measures sub‐section for the definition of “family offer.”) In a secondary analysis, we examined heterogeneity across strata defined by ordinal family offer. We fitted a linear probability model with two‐way interaction terms encoding the extent to which the causal effect of individual offer is modified by family offer levels of zero, one, two, three, four and five or more. We conducted a linear trend test. Finally, we conducted sensitivity analyses which we report in a Supplementary Note.

Models were fitted using robust standard errors, accounting for clustering at the community level. We did not formally adjust for multiple testing as we conducted only three hypothesis tests.

### 2.7 Missing data

We conducted a complete case analysis as only 10/15,675 observations had missing outcome data. All other variables included in the regression models were complete.

### 2.8 Power and sample size

The HITS sample size was calculated to detect a relative reduction of 25% or more in HIV incidence among women aged 15–30 with power exceeding 80% and α = 0.05. Further details have been previously reported [19].

### 2.9 Ethics statement

The Biomedical Research Ethics Committee of the University of KwaZulu‐Natal approved study protocols for the AHRI's population‐based HIV testing platform and HITS intervention (BE290/16 and BFC398/16) [12, 19].

## 3 RESULTS

### 3.1 Participants and network

Of 37,068 residents who met the inclusion criteria for the HITS study, 15,675 participated and 15,665 were included in the analysis (see Figure 1). In total, 5638 participants lived in intervention communities (i.e. communities to whom an incentive was offered) and 10,037 in control communities. Further descriptive results have been previously reported [12].

It was common for participants to have family members in different households (60.4%, 9468/15,675) and different communities (42.2%, 6613/15,675) (see Table 1). However, compared to people living in control communities, people in intervention communities were more likely to have family members who live in an intervention community (85.1%, 4799/5638 vs. 9.4%, 945/10,037). This is because family member households are geographically clustered. Study arms were balanced on age, gender, HIV testing history, network size and proportions of family members in different households and different communities.

**Table 1:** Baseline characteristics

	Control arm (*N* = 10,037)	Incentive arm (*N* = 5638)	Overall (*N* = 15,675)
Age			
15−25	3920 (39.1%)	2292 (40.7%)	6212 (39.6%)
26−35	1690 (16.8%)	922 (16.4%)	2612 (16.7%)
36−45	1194 (11.9%)	679 (12.0%)	1873 (11.9%)
46−55	1247 (12.4%)	695 (12.3%)	1942 (12.4%)
>55	1986 (19.8%)	1050 (18.6%)	3036 (19.4%)
Gender			
Women	6974 (69.5%)	3829 (67.9%)	10803 (68.9%)
Men	3063 (30.5%)	1809 (32.1%)	4872 (31.1%)
Ever tested HIV positive			
Yes	1796 (17.9%)	1029 (18.3%)	2825 (18.0%)
No	6344 (63.2%)	3716 (65.9%)	10060 (64.2%)
Refused	113 (1.1%)	61 (1.1%)	174 (1.1%)
Missing	1784 (17.8%)	832 (14.8%)	2616 (16.7%)
Family network size			
0	290 (2.9%)	134 (2.4%)	424 (2.7%)
1−5	5365 (53.5%)	3029 (53.7%)	8394 (53.6%)
6−10	3064 (30.5%)	1723 (30.6%)	4787 (30.5%)
11−15	961 (9.6%)	539 (9.6%)	1500 (9.6%)
16+	357 (3.6%)	213 (3.8%)	570 (3.6%)
Percentage of family members in different household			
0%	3990 (39.8%)	2217 (39.3%)	6207 (39.6%)
0%−20%	820 (8.2%)	438 (7.8%)	1258 (8.0%)
20%−40%	1484 (14.8%)	909 (16.1%)	2393 (15.3%)
40%−60%	1390 (13.8%)	792 (14.0%)	2182 (13.9%)
60%−80%	1343 (13.4%)	727 (12.9%)	2070 (13.2%)
80%−100%	571 (5.7%)	313 (5.6%)	884 (5.6%)
100%	439 (4.4%)	242 (4.3%)	681 (4.3%)
Percentage of family members in different community			
0%	5831 (58.1%)	3231 (57.3%)	9062 (57.8%)
0%−20%	1237 (12.3%)	655 (11.6%)	1892 (12.1%)
20%−40%	1269 (12.6%)	759 (13.5%)	2028 (12.9%)
40%−60%	803 (8.0%)	486 (8.6%)	1289 (8.2%)
60%−80%	537 (5.4%)	320 (5.7%)	857 (5.5%)
80%−100%	189 (1.9%)	88 (1.6%)	277 (1.8%)
100%	171 (1.7%)	99 (1.8%)	270 (1.7%)
Network treatment (# family members in incentive arm and who have prior study visit)			
0	9092 (90.6%)	839 (14.9%)	9931 (63.4%)
1	551 (5.5%)	1205 (21.4%)	1756 (11.2%)
2	175 (1.7%)	1088 (19.3%)	1263 (8.1%)
3	77 (0.8%)	810 (14.4%)	887 (5.7%)
4	50 (0.5%)	570 (10.1%)	620 (4.0%)
5+	92 (0.9%)	1126 (20.0%)	1218 (7.8%)

**Figure 2:**
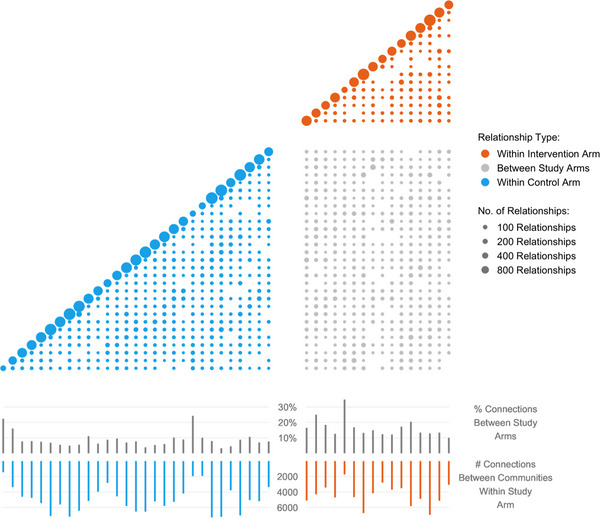
Family connections between communities in HITS study. The top part of the figure is a grid showing the number of family connections within each of the 45 randomization communities on the diagonal, and the number of family connections between each pair of communities below the diagonal. The size of the circles is proportional to the number of connections. Orange and blue bar graphs in the lower part of the diagram show the number of connections across communities but within the intervention arm and control arm, respectively. The grey bar graph shows the proportion of connections across communities in different study arms. The diagram shows that each community was connected to almost every other community through family ties.

Each community had family connections with almost every other community (see Figure 2). Overall, 77% (83,368/107,746) of connections were within communities (as opposed to across them). On average, communities in the control arm had 1947 (56,459/29) connections to individuals in other control communities, whereas communities in the intervention arm had an average of 1682 (26,909/16) connections with individuals in other intervention communities. The proportion of connections that spanned intervention arms was 52% (11,178/21,507) for control communities and 80% (11,178/14,049) for intervention communities.

Two‐thirds (64.7%, 3647/5637) of participants in the intervention arm agreed to take an HIV rapid test, whereas half (50.7%, 5087/10,028) of participants in the control arm agreed, leading to an overall risk difference of 13.6 (95% CI: 12.0−15.3). Of the 8734 participants who consented for an HIV test, HIV test results were recorded for 8700.

### 3.2 Primary analysis

**Figure 3:**
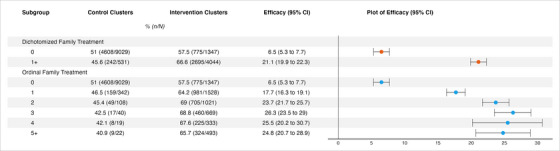
Effect heterogeneity of HITS intervention. The table shows results from the primary analysis (dichotomized family treatment) above and the secondary analysis (ordinal family treatment) below. Efficacy was calculated on the risk difference scale.

We found support for the hypothesis that the effect of the incentive on an individual's HIV testing uptake is augmented by offering incentives to their family members prior to, or at the same time as, them (see Figure 3). Among participants with at least one family member who was offered the incentive, the micro‐incentive increased testing uptake by 21% (95% CI: 19.9−22.3). In contrast, among participants with no family members who were offered the incentive, the micro‐incentive only increased testing uptake by 6.5% (95% CI: 5.3−7.7). The risk difference among the former group is 14.6% higher (95% CI: 9.3−19.9) than among the latter.

### 3.3 Secondary analysis

The strength of the effect of the individual offer increased as more family members received a prior or contemporaneous offer of the incentive, further supporting the main hypothesis. Effect sizes increased from 6.5% (95% CI: 5.3–7.7) among participants with no family members who received the offer to 26.3% (95% CI: 23.5–29.0) among participants with three family members who received it. The effect size of the individual incentive appeared not to change substantially when four (RD: 25.5, 95% CI: 20.2–30.7) or five or more (RD: 24.8, 95% CI: 20.7–28.9) family members received the offer. A linear trend test showed that for each additional family member who was offered the incentive, the risk difference for the effect of the incentive on testing uptake increased by 4.8% (95% CI: 2.4−7.2) on average.

### 3.4 Sensitivity analysis

In the Supplementary Note, we examined the potential impact of selection bias in study participation on the results of the primary analysis. Figure S1 shows a causal directed acyclic graph for HITS study participation. Tables S1 and S3 show study results after conditioning on family size and applying inverse probability of selection weights. Table S2 shows participation rates in AHRI HIV surveillance by age and gender. We conclude that the study findings are robust to selection bias.

## 4 DISCUSSION

The HITS study confirms that a modest community‐wide financial incentive increases the uptake of HIV testing. Our study establishes that an individual offered a financial incentive is more likely to take up testing when family members have received the same offer prior to or at the same time as them. The strength of the effect appears to increase with the count of family members in receipt of the offer.

This finding adds to a growing body of evidence from randomized control trials demonstrating that economic incentives increase the uptake of HIV testing [8, 13, 14, 20–24] and improve clinical cascade outcomes more generally [25]. Past trials show consistent evidence that incentives improve treatment initiation [26, 27], adherence to ART [22, 28–32] and continuation in care [27, 30]. They show mixed evidence that incentives improve linkage to care [26, 27, 33]. Despite their promise as a general‐purpose HIV intervention, however, economic incentives have not been shown to lead to substantial reductions in incidence [34].

Though prior studies are often not explicit about the causal mechanism through which incentives are hypothesized to shape behaviour, several explanations do appear in the epidemiologic literature. Incentives can change the structural environment in which behaviour unfolds (for instance by alleviating poverty); they can affect the price of some behaviour or good, or the income of the recipient in relation to that good or behaviour; and they can intervene on the psychological processes that shape behaviour [35, 36]. In each case, studies commonly assume that the causal chain unfolds entirely *within* individuals and not across them.

There are some notable exceptions. Several trials have shown that incentivizing close social contacts—most commonly romantic [37, 38] or sexual partners [39, 40] or caregivers of children [21, 41]—improves testing uptake. Furthermore, even in the absence of financial incentives, sexual and romantic partnerships have proven a useful conduit through which to deliver HIV testing services [9, 10, 11]. Our study extends these findings to demonstrate the impact of members of the family network in general, suggesting an opportunity to use a wider range of meaningful social relationships to reach individuals living with HIV with testing and other services.

Developing and applying theory that reflects the interdependence of individuals could enable the development of new interventions. For instance, family‐based intervention strategies might be effective at reaching groups which otherwise have low access to health services, such as young people [17, 42]. Because of high youth unemployment in South Africa [17, 18], young people tend to depend on family members for material support [43]. They are likely to be connected with, and therefore, reachable through, members of their family networks. To apply a behavioural economics analysis to this type of intervention, it would be useful to define the concepts of *utility* and *resources* at the group level, to understand decision‐making as a collective (rather than individual) process, and to understand the impact of cognitive biases on this collective process.

We make a novel contribution to the fields of study design and applied causal inference. Our results show empirical evidence for the violation of the assumption of “partial interference” in the context of a large‐scale cluster‐randomized trial [44]. The assumption holds that while individuals within clusters might influence each other's outcomes, individuals across distinct clusters do not. It underpins the interpretation of the difference in average outcomes (comparing intervention and control arms) as an overall treatment effect [44]. When there are substantial connections across clusters, failing to account for them might lead to biased or uninterpretable effect estimates.

It is likely that there are important social relationships that are relevant to HIV testing that were not captured in population surveillance data. This is a limitation of our study. Further research should develop methods to account for missing network data and design new approaches to measuring socio‐centric networks. A further limitation is that we used the assumption of partial interference to calculate standard errors, though we show this assumption to be violated. This was motivated by the fact that network connections are much denser within clusters than they are across them; we do not expect this analytic decision to lead to anti‐conservative estimates of uncertainty. Finally, we did not adjust for multiple testing, though we note that using the Bonferroni correction (i.e. using a nominal Type I error rate of 0.05/3 = 0.017) would not have altered the main conclusions of this study.

A major strength of our findings is that they are not susceptible to homophily bias—bias that arises because of the tendency for people with similar unmeasured characteristics to form relationships based on those characteristics [45]. This is because the study intervention was randomly assigned after the formation of family relationships. A further strength is the applicability of our approach in different settings: it is feasible to conduct a family network analysis using data from any study embedded in the health and demographic surveillance systems of South Africa. Finally, sensitivity analyses show the estimates presented in the main analysis to be conservative.

Understanding humans in the context of their relationships can lead to improvements in population health. There is an urgent need to cultivate robust social network data for epidemiologic analysis—whether by collecting them, constructing them from already collected study data as we did here or connecting passively collected information, such as social media data, with large public health datasets.

## 5 CONCLUSIONS

By combining family network data with data from a field experiment, we showed that network‐based financial incentive programmes for a behavioural health intervention might be more efficient than individual‐based programmes. While the field experiment was conducted in 2018, it is likely that our findings continue to apply in the current context since they are based on long‐standing social relationships among participants. Future HIV testing studies should assess interventions targeted at networks. More generally, public health studies should leverage data on participants’ social networks to generate new insights about population health and to spur on the development of new intervention approaches.

### 5.1 Role of the funding source

Study sponsors had no role in the design, data collection, analysis, interpretation or write‐up of this study, nor did they influence the decisions to submit the results for publication.

## COMPETING INTERESTS

The authors declare no competing interests relating to this manuscript.

## AUTHORS’ CONTRIBUTIONS

KM conducted an analysis and drafted the manuscript. ETT, MTB and LB edited the manuscript. KM developed an analytic plan with ETT, HYK and TB. FT and TB accessed and verified all underlying data. All authors discussed and reviewed the manuscript.

## Supporting information


**Figure S1**: Directed Acyclic Graph for HITS Study Selection.
**Table S1**: Stratum‐specific causal estimates for a financial incentive for HIV Testing.
**Table S2**: Participation in HIV Surveillance by Age and Gender.
**Table S3**: Stratum‐specific causal estimates for a financial incentive for HIV Testing (with inverse probability of selection weighting).

## Data Availability

Study data, including de‐identified participant data and data dictionaries, are available for download from the AHRI Data Repository (https://data.ahri.org/index.php/home) subject to the submission and approval of a study proposal.
