# Supplementary material for: Impact of family networks on uptake of health interventions: evidence from a community‐randomized control trial aimed at increasing HIV testing in South Africa
Source: J Int AIDS Soc. 2023 Aug 20;26(8):e26142. doi: 10.1002/jia2.26142 (PMC10440100; doi:10.1002/jia2.26142)
Supplement: Supplementary file 1 — Figure S1: Directed Acyclic Graph for HITS Study Selection. Table S1: Stratum‐specific causal estimates for a financial incentive for HIV Testing. Table S2: Participation in HIV Surveillance by Age and Gender. Table S3: Stratum‐specific causal estimates for a financial incentive for HIV Testing (with inverse probability of selection weighting). [file JIA2-26-e26142-s001.docx]

**Supplementary Note on Sensitivity Analyses**

Impact of Family Networks on Uptake of Health Interventions: Evidence from a community-randomized control trial aimed at increasing HIV Testing in South Africa

K. Makofane, PhD

H.Y. Kim, PhD

Despite being a cluster-randomized controlled trial, the HITS study could have been vulnerable to selection bias for study participation, potentially due to factors beyond the control of the investigators. This could have implications for bias in the study results (i.e. the extent to which the statistical estimator we used systematically deviates from the ‘true’ underlying causal parameter under investigation), and for generalizability (i.e. the extent to which the ‘true’ underlying causal parameter among study participants accurately reflects the underlying causal parameter among the population from which survey participants were sampled).

Using sensitivity analysis, we examine the likelihood and extent to which the causal estimate reported in our study is unbiased and generalizable.

# Survey Enrollment

To enroll in the HITS study, participants had to go through a three-layer consent process. First, residents were asked if they consent for the study visit. Among those who consented, individuals were asked if they consent to the annual HIV surveillance conducted by AHRI. Finally, among those who consented to the HIV surveillance, participants were asked if they consented for the HITS study.

Because the intervention status of clusters was not concealed from potential participants, there is possibility that their decision to participate in the study may have been influenced by their exposure status. We examine the potential impact of this on the causal estimand under study.

# Bias

Figure S1: Directed Acyclic Graph for HITS Study Selection

## Causal Assumptions

Figure S1 shows a representation of the causal theory underpinning our analysis. Solid arrows represent causal pathways we assume exist, and the dotted arrow shows the causal pathway under investigation. Circles represents variables, and squares represent variables on which the analysis is conditioned.

Individual willingness to take a rapid HIV test ($W$) is assumed to be caused by Family Intervention Status ($F$ – a variable measuring whether the participant’s family members received the offer the incentive), family size ($S$), and other unmeasured variables ($U$).

Individual offer of the financial incentive ($A$) is assumed to be caused exclusively by the intervention status of the community ($C$). Family intervention status ($F$) is caused by $C$ as well as $S$.

We hypothesize that the individual receipt of the offer of an incentive ($A$), causes willingness to take a rapid HIV test ($W$). We assume that the effect of all prior variables on $Y$ is mediated exclusively by $W$.

Finally, we assume that consent for HIV surveillance ($C_{s}$) is caused by $W$, among other variables. Consent for the HITS study ($C_{H}$) is also caused by $W$, among other variables. The analysis is conditioned on $C_{s}$ and $C_{H}$ since we only observe participants who consented to both HIV surveillance, and HITS. Since we examine the $A\to Y$ relationship within levels of $F$, the analysis is conditioned on $F$ as well.

## Implications for Bias

Under the assumptions that

1. people who desire to take an HIV test are more likely to consent to the HIV surveillance and to the HITS study and,
2. people who know that they are in the intervention arm are more likely to participate in the study

we would find that the effect of one’s own intervention status on one’s likelihood to test is biased in the negative direction. This would be a result of collider stratification bias as defined through the rules of D-Separation (Hernán and Robins 2018).

i.e. According to the Directed Acyclic Graph (DAG) shown in Figure S1, there are two backdoor paths connecting one’s individual intervention status (A) with the study outcome which is uptake of rapid HIV testing (Y): $A\to\boxed{C_{H}}\leftarrow W\to Y$ and $A\to\boxed{C_{S}}\leftarrow W\to Y.$These backdoor paths would tend to bias the measured relationship between $A$ and $Y$ in the negative direction.

However, since we examine the relationship between individual exposure status ($A$) and the outcome ($Y$) within strata defined by family intervention status ($F$), we open the backdoor paths $A\leftarrow C\to\boxed{F}\leftarrow S\leftarrow U\to W\to Y$ and $A\leftarrow C\to\boxed{F}\leftarrow S\to W\to Y$. To close these backdoor paths, it is sufficient to condition on family size ($S$). Therefore, after adjusting for family size, the only backdoor paths connecting exposure and outcome bias the effect estimates within strata defined by F. These paths will tend to bias the relationship between $A$ and $Y$ in the negative direction.

We note that the causal estimand of interest is not the stratum-specific effects of $A$ on $Y$, it is the difference between the effect sizes. Bias in the causal estimand of interest would occur if the effect estimate in one stratum was biased to a different extent than the effect estimate in the other stratum.

Defining $\delta$ as the causal estimand of interest, $\delta_{-}$ as the effect of the individual incentive on HIV Testing uptake among those whose family members were not offered the incentive, and $\delta_{+}$ as the effect among those whose family members were offered the incentive, we have:

$$\delta\equiv\delta_{+}-\delta_{-}$$

Further defining $\hat{\delta}$, $\hat{\delta}_{+}$, and $\hat{\delta}_{-}$ as the statistical estimators for parameters $\delta$, $\delta_{+}$, and $\delta_{-}$, respectively:

$$Bias\left[ \hat{\delta} \right]=\delta-E[\hat{\delta}]$$

$$=\delta_{+}-\delta_{-}-E[\hat{\delta}_{+}-\hat{\delta}_{-}]$$

$$=Bias\left[ \hat{\delta}_{+} \right]-Bias\left[ \hat{\delta}_{-} \right]$$

Say we estimated $\hat{\delta}$ as having a positive point estimate with confidence interval [$ci_{lb}-ci_{ub}$] where $ci_{lb},ci_{ub}>0$, and we believed that this estimate is possibly biased by collider-stratification. Following VanderWheele et al. (2017), we would say the bias explains away the causal estimate if removing the bias caused the confidence interval to shift so that it includes 0. i.e. The causal estimate is explained away by bias if:

$$ci_{lb}-Bias\left[ \hat{\delta} \right]\leq0$$

$$Bias\left[ \hat{\delta}_{+} \right]-Bias\left[ \hat{\delta}_{-} \right]\geq ci_{lb}$$

Table S1, below, shows the stratum specific effect sizes, and the difference in effect sizes both in the original analysis (upper half of the table) and an analysis which is conditioned on family size (lower half of the table). Referring to the conditional analysis, $\hat{\delta}$ is estimated as 17.5 with a 95% confidence interval of (7.3 to 27.7). For the main conclusion of the study to be explained away by the bias induced by the open backdoor paths described above, the bias of the effect size among those with no family members who were offered the incentive would have to be larger (in absolute terms on the linear scale) by 7.3 percentage points (the lower bound of the confidence interval) than the bias among those with family members who received the offer.

Table S1: Stratum-specific causal estimates for a financial incentive for HIV Testing

|  | **Family Not Offered Incentive** | **Family Offered Incentive** | **Difference in Effect Sizes** | **p** |
| --- | --- | --- | --- | --- |
| **Unadjusted** | | | | |
| Individual Not Offered Incentive | 51.0 (1.3) | 45.6 (2.3) |  |  |
| Individual Offered Incentive | 57.5 (2.0) | 66.6 (1.2) |  |  |
| Effect of Individual Incentive | 6.5 (1.5 to 11.5) | 21.1 (15.7 to 26.5) | 14.6 (8.1 to 21.0) | 0.000 |
| p | 0.012 | 0.000 |  |  |
| **Adjusting for Family Size** | | | | |
| Individual Not Offered Incentive | 51.1 (1.2) | 46.1 (3.3) |  |  |
| Individual Offered Incentive | 53.8 (3.5) | 66.2 (1.2) |  |  |
| Effect of Individual Incentive | 2.6 (-5.7 to 10.9) | 20.1 (13.2 to 27.0) | 17.5 (7.3 to 27.7) | 0.001 |
| p | 0.536 | 0.000 |  |  |

# Generalizability

Table S2: Participation in HIV Surveillance by Age and Gender

|  | **Refused HIV Surveillance**  **(N=9907)** | **Participated in HIV Surveillance**  **(N=15675)** | **Overall (N=25582)** | **P-value** |
| --- | --- | --- | --- | --- |
| **Gender** |  |  |  |  |
| Women | 5133 (51.8%) | 10803 (68.9%) | 15936 (62.3%) | <0.001 |
| Men | 4774 (48.2%) | 4872 (31.1%) | 9646 (37.7%) |  |
| **Age** |  |  |  |  |
| Mean (SD) | 34.2 (18.1) | 35.3 (19.6) | 34.9 (19.0) | <0.001 |
| Median [Min, Max] | 30.3 [8.78, 95.5] | 30.7 [10.1, 99.7] | 30.5 [8.78, 99.7] |  |
| Missing | 2 (0.0%) | 2 (0.0%) | 4 (0.0%) |  |

As shown is Table S2, women were more likely than men to consent to HIV surveillance, and older people were more likely than younger people to consent. To the extent that age or sex modify the relationship between the study exposure and outcome, these differences in study participation might indicate that study results are not generalizable to the underlying population.

To bring the study sample closer to the underlying population, we conducted the analyses whose results are shown in Table S1 again, this time, using inverse probability of selection weights. The weights were constructed using a logistic regression model with study participation as outcome, gender as a categorical variable, and age as a degree-2 polynomial. The results, shown in Table S3 below. The difference in effect sizes between strata decreases by 0.1 percentage points, and the lower bound decreases by 0.2 percentage points.

Table S3: Stratum-specific causal estimates for a financial incentive for HIV Testing (with inverse probability of selection weighting)

|  | **Family Not Offered Incentive** | **Family Offered Incentive** | **Difference in Effect Sizes** | **p** |  |
| --- | --- | --- | --- | --- | --- |
| **Unadjusted** | | | | | |
| Individual Not Offered Incentive | 51.4 (1.4) | 45.7 (2.5) |  |  |  |
| Individual Offered Incentive | 58.0 (1.9) | 66.7 (1.2) |  |  |  |
| Effect of Individual Incentive | 6.6 (1.9 to 11.4) | 21.0 (15.8 to 26.2) | 14.4 (8.0 to 20.9) | 0.000 |  |
| p | 0.006 | 0.000 |  |  |  |
| **Adjusting for Family Size** | | | | | |
| Individual Not Offered Incentive | 51.5 (1.2) | 46.4 (3.3) |  |  |  |
| Individual Offered Incentive | 54.4 (3.3) | 66.3 (1.3) |  |  |  |
| Effect of Individual Incentive | 2.9 (-4.7 to 10.4) | 19.9 (13.4 to 26.3) | 17.0 (7.1 to 26.9) | 0.001 |  |
| p | 0.454 | 0.000 |  |  |  |

# Conclusions

Conditioning on family size yields a larger causal estimate than the one presented as the main study result. The result is, therefore, conservative. Furthermore, using inverse probability of selection weights to adjust for potential selection bias does not materially change the causal estimate.

It is possible that the main estimate is biased by collider-stratification bias in each stratum defined by family intervention status. For this to explain away the main study finding, it would have to be the case that the difference in the magnitude of the bias in each stratum is at least 7.3 percentage points. We therefore conclude that the main study findings are robust to this type of bias.

# References

Hernán, Miguel, and James M. Robins. 2018. *Causal Inference*. Chapman & Hall/CRC Monographs on Statistics & Applied Probability. Chapman & Hall/CRC.

VanderWeele, Tyler J, and Peng Ding. 2017. “Sensitivity Analysis in Observational Research: Introducing the E-Value.” *Annals of Internal Medicine* 167 (4): 268–74.
